# Severity of Psychotic‐Like Experiences in Help‐Seeking German Adolescents: An Exploration of Psychosocial Predictors and Psychological Treatment Outcomes

**DOI:** 10.1111/eip.70084

**Published:** 2025-08-03

**Authors:** Phuong Mi Nguyen, Sandra Abrantes‐Diaz, Sören Friedrich, Karen Krause, Silvia Schneider, Mar Rus‐Calafell

**Affiliations:** ^1^ Mental Health Research and Treatment Centre Faculty of Psychology, Ruhr‐Universität Bochum Bochum Germany; ^2^ German Centre of Mental Health (DZPG), Partner Site Bochum/Marburg, Ruhr‐Universität Bochum Bochum Germany

**Keywords:** cognitive behavioural therapy, psychosocial factors, psychotic‐like experiences, transdiagnostic approach

## Abstract

**Introduction:**

Psychotic‐like experiences (PLEs) occur transdiagnostically in young people and are linked to an increased risk of severe psychopathology in adulthood. However, the psychosocial factors influencing PLEs severity and their distribution across diagnoses remain under‐researched. Updated knowledge of these aspects is crucial for clinical understanding and treatment. This study aims to expand existing research by investigating the prevalence and psychosocial factors associated with PLEs in help‐seeking adolescents, examining differences in their severity across diagnoses and multimorbidity, and exploring preliminary changes following routine cognitive behavioural therapy (CBT).

**Methods:**

This observational, non‐controlled study included 275 adolescents attending a routine mental health service for young people in Germany. Of the full sample, 161 participants completed a full course of CBT.

**Results:**

At baseline, 87% reported at least one PLE, and 54.91% reported three or more. The most distressing experiences included thought broadcasting, paranoia, mind reading, and auditory hallucinations. The number of diagnoses did not affect PLEs severity, but the presence of self‐reported childhood physical neglect and jumping to conclusions did. Among those who completed CBT, results showed reductions in PLEs severity over time across diagnostic groups.

**Discussion:**

This study highlights the high prevalence of transdiagnostic PLEs in help‐seeking adolescents. Among psychosocial predictors, trauma and cognitive biases were particularly relevant and should be addressed in psychotherapy. The observed improvements in PLEs severity following CBT, irrespective of the primary presenting problem, suggest promising avenues for transdiagnostic therapeutic strategies.

## 1 Introduction

Psychotic‐like experiences (PLEs) refer to subclinical psychotic symptoms like believing to be spied upon or hearing/seeing things without external corresponding stimuli. Experiencing PLEs during childhood and adolescence has been associated with a higher risk of developing psychosis and other mental health disorders (Healy et al. 2019). Persistence, associated distress and psychopathology of these experiences are important predictors of transition to a clinical disorder and need for mental health care (Connell et al. 2016; Maijer et al. 2018; Yates et al. 2019). PLEs have also been associated with multimorbidity, suicidality (Kelleher et al. 2014), and severity of psychopathology (Palstra et al. 2024) in help‐seeking young people, highlighting the need to screen for these experiences to improve diagnosis and prevent under‐treatment of comorbid psychopathology.

Considering the strength of this evidence, relevant clinical guidelines recommend that treatment should be offered where appropriate for childhood and adolescence presentations of PLEs when these are distressing or interfering in the young person's social functioning (NICE 2013; van der Gaag et al. 2013). In Germany, treatment recommendations for children and adolescents with psychosis are currently integrated within the adult S3 guidelines for schizophrenia (Deutsche Gesellschaft für Psychiatrie und Psychotherapie, Psychosomatik und Nervenheilkunde, DGPPN 2019).

Current treatment recommendations for young people with PLEs are therefore based on research in adults with psychosis, and on the assumption that similar psychological processes underlie these experiences and their associated distress. Psychological cognitive models of psychosis have proposed that cognitive, behavioural, emotional, and social processes are crucial for the development and persistence of distressing positive symptoms in adults (Garety et al. 2001). Cognitive Behavioural Therapy for Psychosis (CBTp) draws upon these existing models (Morrison and Wood 2023). Factors like childhood trauma and adversity, cognitive biases and emotional processes have been pointed out as determinants for the transition from subclinical symptoms to a clinical disorder (Connell et al. 2016; Maijer et al. 2018; Yates et al. 2019). However, only a few recent studies have provided evidence supporting at least partial mirroring of this model in youth experiencing PLEs. Cognitive biases (e.g., jumping to conclusions, JTC) have been found to predict the presence and severity of PLEs in different studies (Gin et al. 2021; Ames et al. 2014; Hassanali et al. 2015; the last two studies focus on the same sample). Limited evidence supporting the role of childhood trauma and adversity (Kelleher et al. 2014), and negative emotional processes (Ames et al. 2014), has also been provided. However, except for the study by Gin et al. (2021), evidence for the association of these psychosocial processes and the severity of PLEs comes mainly from studies conducted with young adolescents (8–14 years old) seeking help and in English‐speaking countries.

Germany has a long tradition of early detection services (Maric et al. 2019) and highly specialised detection protocols. However, psychosis treatment is limited and mainly offered to adults with well‐established diagnoses and in inpatient clinics, less in an outpatient setting (DGPPN 2019; Schlier et al. 2017). This approach involves making evidence‐based treatment decisions mainly for individuals with fully developed, prototypical, and relatively late‐stage syndromes, typically managed within adult specialised service systems. However, these rigid taxonomies often fail to address emerging and early‐stage illness, continuity, and comorbidity, and do not align well with transdiagnostic staging models proposed for youth mental health (Shah et al. 2020). Although a national strategy for the implementation of integrated youth mental health care is yet to be established, there are few emerging transdiagnostic and interdisciplinary initiatives that aim to transform current clinical practices for early psychosis in Germany (Bechdolf et al. 2024; Schultze‐Lutter et al. 2015).

Despite the clinical relevance of PLEs for young people's mental health, research on key associated psychological factors, diagnostic differences, and the potential under‐treatment of PLEs in help‐seeking individuals remains limited. This lack of evidence is hindering the establishment of consensual clinical practices when attending to young people.

The present study aims to contribute new evidence toward a better understanding of PLEs and their clinical management in young clinical populations. To our knowledge, it is the first study to investigate the transdiagnostic prevalence and psychological predictors of PLE severity in a large help‐seeking adolescent clinical sample in Germany. Importantly, it also examines the impact of CBT on the severity of PLEs across various diagnoses, as delivered within a routine mental health service for young people. This study had three main objectives: (1) to assess the prevalence of PLE and psychosocial predictors of severity in a sample of adolescents seeking help; (2) to investigate differences in PLE severity across mental health diagnoses and associated multimorbidity; and (3) to explore the potential impact of evidence‐based CBT on PLE severity across diagnoses at post‐treatment.

## 2 Materials and Methods

### 2.1 Participants

The study follows a non‐controlled, observational design, including seeking‐help adolescents at the outpatient clinic of the Mental Health Research and Treatment Centre of the Ruhr‐University in Bochum (MHRTC), which follows regulatory insurance procedures of the German health system. The study was part of the routine clinical diagnosis assessment at the MHRTC, for which informed (parental) consent was obtained at the beginning of therapy. Before undergoing therapy in the outpatient clinic, all clients (or their parents) sign a treatment contract and give consent to complete questionnaires during therapy, which are also used for scientific purposes. The study was reviewed and approved by the local Ethics Committee of the Faculty of Psychology at the Ruhr‐University Bochum (Votum: 431).

Inclusion criteria were age between 12 and 18 years and sufficient proficiency in German to participate in assessment and therapy. Exclusion criteria included a diagnosis of intellectual disability, autism spectrum disorder, or psychosis.

### 2.2 Procedure

Data was extracted from structured clinical interviews (Kinder‐DIPS; Schneider et al. 2017) and self‐reported questionnaires, which are part of the routine clinical assessment in the MHRTC. For the present study, we added questionnaires on trauma, cognitive bias and PLEs as part of this routine assessment (as PLEs were not systematically assessed before the start of the present study). Participants completed assessments at regular intervals before, during, and after therapy as part of the outpatient clinic's standard procedures for quality assurance and treatment monitoring. Therapists providing care were trained in cognitive behavioural therapy (CBT) and had a minimum of 1 year of clinical experience. Final diagnostic decisions were reviewed and approved by experienced supervisors.

### 2.3 Measures

The Kinder‐DIPS is a structured diagnostic interview designed to assess mental disorders in children and adolescents (*Diagnostisches Interview bei psychischen Störungen im Kindes‐ und Jugendalter*; Schneider et al. 2017). It is an open‐access tool that guides interviewers in identifying therapy‐relevant mental disorders in accordance with the Diagnostic and Statistical Manual of Mental Disorders, German version of DSM‐5 (Falkai and Wittchen 2015). It consists of an interview with the client and caregiver and has a high interrater reliability between the child and parent versions (Neuschwander et al. 2013). For simplicity, the term *anxiety disorders* is used for neurotic, stress‐related, and somatoform disorders, the term *mood disorders* is used for affective disorders and *behavioural disorders* is used for behavioural syndromes and conduct disorders. Please refer to the Supporting Information for a detailed list of diagnoses in each group.

The Unusual Experiences Questionnaire (UEQ; Ames et al. 2014) is a self‐report measure comprising nine items, each assessing a specific psychotic‐like experience. Five items were adapted by Laurens et al. (2012) from the Diagnostic Interview Schedule for Children (Costello et al. 1982), while the remaining four items were developed to capture a broader range of hallucination‐ and delusion‐like phenomena (Laurens et al. 2011). The internal consistency of the UEQ was acceptable, with a Cronbach's alpha of 0.78 for the current sample.

The Davos Assessment of the Cognitive Biases Scale (DACOBS; van der Gaag et al. 2013) was included to assess the presence of cognitive biases. This self‐reported questionnaire measures 4 cognitive biases specific to positive symptoms of psychosis (JTC, belief inflexibility, selective attention for threat and external attribution bias), 2 cognitive limitations (social cognition problems and subjective cognitive problems) and avoidance behaviour. For this study, the validated 18‐item version (scored on a 7‐point Likert scale with a 2‐week period) was used (Gawęda et al. 2018). The internal consistency of the DACOBS was good, with a Cronbach's alpha of 0.89 for the total score and values ranging from 0.73 to 0.81 across the subscales.

Childhood trauma and adversity were assessed using the Childhood Trauma Screener (CTS), the German short form of the Childhood Trauma Questionnaire (CTQ), consisting of five items (Grabe et al. 2012). Participants rated experiences of emotional, physical, and sexual abuse, as well as emotional and physical neglect, on a 5‐point Likert scale ranging from 1 (“not at all”) to 5 (“very frequently”). Internal consistency for the CTQ was deemed acceptable in this study, as indicated by a Cronbach's alpha coefficient of 0.74.

The Strengths and Difficulties Questionnaire (SDQ; Klasen et al. 2003) is a 25‐item self‐report screening measure of general childhood psychopathology. It includes five subscales, each consisting of 5 items: emotional symptoms, peer relationship problems, conduct problems, hyperactivity–inattention, and prosocial behaviour. Each SDQ item is rated on a 3‐point scale. The SDQ demonstrated acceptable internal consistency, with a Cronbach's alpha of 0.71 for the total score and subscale alphas ranging from 0.70 to 0.79.

### 2.4 Statistical Analysis Plan

Statistical analysis was conducted in R Studio for Windows, Version 2022.12.0.353. Descriptive statistics are presented using frequency and percentages for categorical variables; mean (*M*) and standard deviations (SD) for continuous variables. Welch ANOVA and non‐parametric Wilcoxon tests were applied when violations of homoscedasticity and normality were encountered. A generalised mixed model analysis (GLMM) was applied to explore the predicting power of psychosocial variables on PLEs severity. Differences in severity between diagnoses were assessed using ANOVA, while linear regression was applied to examine the relation between severity and multimorbidity. GLMM was also used to explore changes in PLEs severity after CBT.

## 3 Results

At baseline, 275 young clients were included in the study, with a mean age of 15.25 years (SD = 1.86); 74.91% of the participants were female. Among these individuals, 206 meet criteria for a mental health disorder: anxiety disorders (50%), mood disorders (26.7%), or behavioural disorders (23.3%); 161 of these participants completed a full course of CBT therapy: *n* = 102 participants had their post‐treatment assessment after at least 12 sessions, and *n* = 59 after 24 sessions. At baseline, only around one fifth of the participants (18.6%) reported no previous experience of trauma. Among the various traumatic experiences, *emotional neglect* emerged as the most prevalent one. Table 1 lists further demographics and clinical characteristics.

**TABLE 1:** Demographics and clinical characteristics (*N* = 275).

Variable	*M* (SD)
Age	15.25 (1.86)
Gender*	
Male	69 (25.09)
Female	206 (74.91)
Primary diagnosis*	
Mood disorders	55 (20)
Anxiety disorders	103 (37.45)
Behavioural and emotional disorders	48 (17.45)
Trauma*	
Emotional neglect	123 (70.93)
Emotional abuse	98 (56.4)
Physical neglect	53 (30.18)
Physical abuse	43 (24.42)
Sexual abuse	30 (16.86)
Cognitive biases	
Jumping to conclusions	7.46 (3.12)
Belief inflexibility	3.6 (1.83)
Attention to threat	15.1 (4.81)
External attribution	6.37 (2.59)
Strength and difficulties questionnaire	
Emotional symptoms	6.86 (2.43)
Conduct problems	2.23 (1.64)
Hyperactivity	4.98 (2.24)
Peer relationship problems	3.89 (2.09)
Prosocial	7.98 (1.92)

*Note:* *Variables reported by *N* (%).

### 3.1 Prevalence, Distress and Severity of PLEs

Almost 87% of participants reported at least one PLE at baseline, and 54.91% reported three or more different PLEs. “*Persecutory ideation*” was the most reported PLE (69.45%), followed by “*being able to read others' thoughts*” (55.27%), and feelings that people “*can read my mind*” (34.55%). In addition, “*hearing voices when no one was around*” was reported by 29.82% of the individuals. “*Persecutory ideation*” was the most distressing PLE. Clients who did not report any PLEs were excluded from this estimation. Table 2 depicts the percentage of young people endorsing each PLE.

**TABLE 2:** Prevalence of endorsed PLE at baseline assessment (*N* = 275).

UEQ item	True	No distress	A bit	Quiet a lot	Very much
1. Some people believe that their thoughts can be read. Have other people ever read your thoughts?	**95** **(34.55)**	41 (14.91)	33 (12)	15 (5.45)	5 (1.82)
2. Have you ever believed that you were being sent special messages through the television?	67 (24.36)	38 (13.82)	19 (6.91)	7 (2.55)	2 (0.73)
3. Have you ever thought that you were being followed or spied on?	**191** **(69.45)**	23 (8.36)	85 (30.91)	58 (21.09)	25 (9.09)
4. Have you ever heard voices that other people could not hear?	**82** **(29.82)**	18 (6.55)	35 (12.73)	19 (6.91)	10 (3.64)
5. Have you ever felt that you were under the control of some special power?	38 (13.82)	10 (3.64)	15 (5.45)	10 (3.64)	3 (1.09)
6. Have you ever known what another person was thinking even though that person wasn't speaking?	**152** **(55.27)**	122 (44.36)	22 (8)	5 (1.82)	3 (1.09)
7. Have you ever felt as though your body has been changed in some way that you could not understand?	53 (19.27)	15 (5.45)	26 (9.45)	7 (2.55)	5 (1.82)
8. Do you have any special powers that other people don't have?	25 (9.09)	16 (5.82)	6 (2.18)	1 (0.36)	0 (0)
9. Have you ever seen something or someone that other people could not see?	64 (23.27)	23 (8.36)	16 (5.82)	16 (5.82)	9 (3.27)
Total endorsing at least one PLE	118 (42.91)				
Total PLE severity score *M* (SD)	21.55 (11.59)				

*Note:* Results are presented as *N* (%), except “total PLE severity score”. Items in bold are those with > 25% of the sample affirmative endorsing.

### 3.2 Psychosocial Predictors of Severity

**FIGURE 1:**
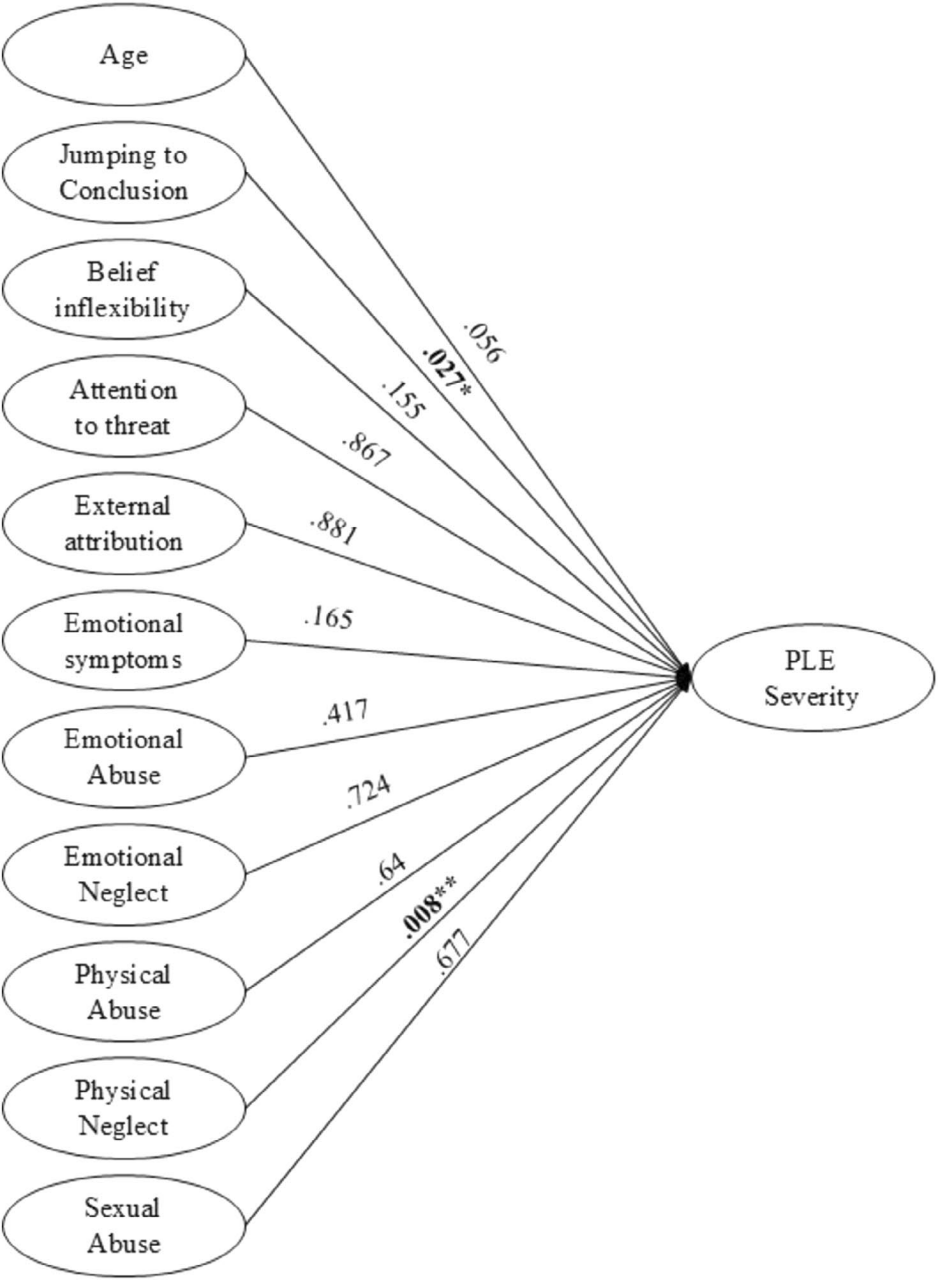
Predictors of the severity of psychotic‐like experiences.

The regression analysis indicated that JTC (*ß*_1_ = 0.031, SE = 0.014, *z* = 2.214, *p* = 0.027) and physical neglect (*ß*_2_ = 0.205, SE = 0.077, *z* = 2.662, *p* = 0.008) were significant predictors of higher PLEs severity. The model explained approximately 79.5% of the variance (*R* ^2^ = 0.795; AIC = 1084.5, deviance = 1060) (see Table 3 and Figure 1).

**TABLE 3:** Regression model PLE severity.

Variable	PLE severity score			
	*ß*	SE	*z*	*p*
Age	−0.042	0.021	−2	0.056
Fixed effects				
Cognitive biases				
Jumping to conclusion	0.031	0.014	2.214	0.027*
Belief inflexibility	0.037	0.026	1.423	0.155
Attention to threat	0.002	0.012	0.167	0.867
External attribution	−0.003	0.02	−0.15	0.881
Emotional symptoms (SDQ)	0.025	0.018	1.389	0.165
Trauma (CTS)				
Emotional abuse	0.073	0.09	0.811	0.417
Emotional neglect	0.03	0.085	0.353	0.724
Physical abuse	0.043	0.092	0.467	0.64
Physical neglect	0.205	0.077	2.662	0.008**
Sexual abuse true	−0.016	0.101	−0.416	0.677

### 3.3 Distribution Across Diagnoses and Multimorbidity

The proportion of individuals reporting PLEs was similar across the three main diagnostic groups. Table 4 depicts the distribution of the number of PLEs and the severity scores for each diagnostic group.

**TABLE 4:** Number of PLE by diagnosis group.

	Mood disorders (*N* = 55)	Anxiety disorders (*N* = 103)	Behavioural disorders (*N* = 48)
None*	8 (14.54)	13 (12.62)	4 (8.33)
One or more*	47 (85.45)	90 (87.37)	44 (91.67)
One	9 (16.36)	19 (18.45)	9 (18.75)
Two	5 (4.85)	16 (15.53)	9 (18.75)
Three	33 (60)	55 (53.4)	26 (54.17)
PLE_Severity Score_ M (SD)	21.81 (10.62)	20.5 (10.38)	22.5 (12.93)

*Note:* *Variables reported by *N* (%).

A total of *n* = 159 individuals had a single clinical diagnosis, while *n* = 46 were diagnosed with two or more clinical diagnoses (multimorbidity). Results from the regression model indicated that participants with a single diagnosis did not report significantly lower PLEs severity (*M* = 21.1, SD = 10.7) compared to those with multiple diagnoses (*M* = 22.4, SD = 12.5) (*p* > 0.05).

### 3.4 PLEs Severity Changes After CBT

Exploratory analyses were performed to assess PLEs severity changes after CBT for the three diagnosis groups. Results from the GLMM analysis revealed a statistically significant main effect of time (*ß* = −0.203, SE = 0.042, *p* < 0.001), indicating a reduction in PLE severity over time across all groups. Significant interaction effects between time and diagnosis group further showed that the anxiety disorders group experienced a greater reduction in PLE severity compared to both the Mood Disorders group (*ß* = 0.163, SE = 0.068, *p* = 0.017) and the Behavioural Disorders group (*ß* = 0.142, SE = 0.066, *p* = 0.032). Means and standard deviations of PLEs severity scores at pre‐ and post‐CBT by diagnosis group are presented in Table 5.

**TABLE 5:** Pre‐ and post‐PLE severity score across diagnosis groups.

	Baseline		Follow‐up	
	*M*	SD	*M*	SD
Mood disorders	21.81	10.62	20.42	15.85
Behavioural disorders	22.5	12.93	20.79	12.30
Anxiety disorders	20.5	10.83	17.46	7.94

## 4 Discussion

This is, to our knowledge, the first study examining prevalence rates, psychosocial predictors, prevalence distribution across different diagnoses, and multimorbidity of PLEs in a German adolescent clinical sample. The results indicate that experiencing PLEs is prevalent in this young age group and occurs transdiagnostically. Adolescents with multiple clinical diagnoses did not differ from those with a single diagnosis in terms of PLEs severity. Additionally, JTC bias and trauma (specifically physical neglect) were significantly associated with PLEs severity. Interestingly, clients showed a reduction in PLEs severity after CBT treatment, with greater improvements in clients with anxiety disorders.

Regarding the first study aim, results indicate that the most prevalent PLEs (each reported by more than 25% of the sample) were experiences of thoughts being read, paranoia, mind reading, and auditory hallucination‐like phenomena. These findings align with previous research on younger populations (Kelleher et al. 2014; Laurens et al. 2012), which similarly identified auditory hallucination–like experiences and paranoid thoughts as the most commonly reported PLEs. In the present study, approximately 87% of clients reported experiencing at least one PLE at baseline. This is consistent with prior research. Laurens et al. (2012) reported that two‐thirds of children aged 9–11 had experienced at least one PLE, while Ames et al. (2014) found that 85% of their sample endorsed similar experiences.

Goodness of fit of the cognitive model of psychosis (Garety et al. 2001; Garety et al. 2007) appears limited in the present study. Significant associations between PLEs severity and the proposed psychosocial factors were only found for the JTC bias and physical neglect. Other reasoning biases, emotional difficulties, and types of trauma were not significantly associated with PLE severity. Our results only partially support those provided by previous studies exploring the contribution of cognitive and psychosocial factors to PLEs (Ames et al. 2014; Livet et al. 2020). Taken together, the results highlight JTC as a key factor consistently associated with PLE severity across young non‐clinical and clinical population (Ames et al. 2014; Gin et al. 2021), individuals at risk of developing psychosis (Kelleher and Cannon 2011), and those with clinical psychosis (Dudley et al. 2016; So et al. 2016). This consistent research finding endorses the application of mechanism‐based interventions targeting this reasoning bias, which have been shown to reduce cognitive biases, delusional conviction, and distress in individuals with psychosis (Garety et al. 2020; Garety et al. 2021) and have also demonstrated promising results in enhancing positive appraisals of PLEs in adolescents (Radez et al. 2024).

Although many individuals reported experiences of childhood trauma, only physical neglect was significantly associated with PLEs severity in the present study. Nevertheless, other types of traumatic experiences have been positively associated with PLEs severity in previous adolescent studies (Ames et al. 2014; Gin et al. 2021). A plausible explanation for the inconsistency with previous studies is that the Childhood Trauma Scale (CTS) may not adequately capture all relevant types of trauma in this age group, such as bullying (Kranhold et al. 2021). Previous studies have identified an association between various forms of bullying and the development of PLEs (Catone et al. 2017). Additionally, it is possible that trauma assessment occurred too early in therapy, as research indicates that, particularly in young people, disclosure of traumatic experiences is more likely during in‐person therapy sessions once a therapeutic alliance has been established (Howard et al. 2022).

Regarding our second aim, results showed no differences in the distribution of PLEs across diagnostic groups, and PLEs severity was not associated with multimorbidity. Interestingly, over 50% of the young patients reported experiencing three or more PLEs across all three diagnostic categories. Since the risk of suicide and increased severity of psychopathology have been associated with PLEs (Kelleher et al. 2014; Palstra et al. 2024), clinicians should pay additional attention and consideration to psychopathology and risks beyond the primary diagnosis to inform the person's treatment plan.

Finally, the exploratory analysis of CBT outcomes showed improvement in PLEs severity at post‐treatment in all three diagnostic categories. Results showed that clients with anxiety disorders showed greater improvements in PLEs severity compared to those with depressive or behavioural disorders; however, the study was not powered to detect group differences. It is important to note, however, that CBT was tailored to the clients' primary diagnoses and was not specifically designed to target distressing PLEs.

A possible explanation for this finding is that CBT for anxiety and emotional disorders specifically targets dysfunctional cognitions and cognitive biases, which are core psychological mechanisms underlying anxiety disorders (Rapee et al. 2023). However, it remains unclear whether improvements in PLEs severity could be enhanced by introducing important components of CBT for psychosis such as normalisation of experiences or specific training to reduce JTC (regardless of the primary diagnosis, but when severity of PLEs is detected). In line with this approach, recent evidence has emerged for novel transdiagnostic CBT‐based psychotherapies for distressing PLEs in children and adolescents (Jolley et al. 2018). Nonetheless, the first main step remains in the early detection of PLEs, and the clinicians' recognition and acknowledgment of associated distress in young individuals, irrespective of primary diagnosis.

## 5 Limitations

The present results should be interpreted with caution due to several limitations. First, as noted earlier, the choice of trauma questionnaire may have limited the detection of relevant trauma types. Second, the CBT outcomes reported here focus solely on pre‐post changes in PLEs and should be considered alongside broader clinical effects and improvements in diagnosis‐specific symptoms. Third, the MHRTC is a university outpatient clinic where psychotherapists and trainees have direct access to ongoing education and supervision from highly experienced clinicians, resources that may not be consistently available in other routine outpatient services in Germany. Finally, a limitation of the present study is the absence of a control group. As a result, the observed pre‐post changes following CBT cannot be confidently attributed to the intervention itself. It remains unclear whether similar changes would have occurred without any treatment. To establish causality, a sufficiently powered randomised controlled trial would be required.

## 6 Conclusions

These findings support standardisation and wider consensus of PLEs screening in help‐seeking adolescents, due to its prevalence and potential associated distress. The findings in this study imply that working with CBT without especially targeting PLE could help young people in reducing PLE associated distress. It also seems plausible that small adaptations of CBT (e.g., normalisation of PLE, targeting emotional problems or reasoning bias) for affective or anxiety disorders in children and adolescents could be sufficient to achieve a clinically relevant change in concomitant distressing PLE. The transdiagnostic approach adopted in this study aligns with emerging youth services in Germany, such as *Soulspace* in Berlin (Bechdolf et al. 2024), which also aim to implement youth mental health care based on transdiagnostic staging models (Shah et al. 2020). Following state‐of‐the‐art models and updated principles to improve youth mental health (McGorry et al. 2024), our team at the MHRTC has also started a specialised psychological consultation service for young people suffering from distressing PLEs, regardless of meeting criteria for a clinical diagnosis.

## Ethics Statement

The authors assert that all procedures contributing to this work comply with the ethical standards of the relevant national and institutional committees on human experimentation and with the Helsinki Declaration of 1975, as revised in 2008. The study was reviewed and approved by the local Ethics Committee of the Faculty of Psychology at the Ruhr‐University‐Bochum (Votum: 431) as part of the standard diagnostic examination procedure at the Mental Health Research and Treatment Centre in Bochum.

## Conflicts of Interest

The authors declare no conflicts of interest.

## Supporting information


**Data S1:** Diagnoses in the categories.

## Data Availability

The data supporting the findings of this study are available from the corresponding author upon reasonable request. Due to the sensitive nature of psychotherapy data and participant confidentiality, the dataset is not publicly available to comply with ethical and privacy requirements.
