# Supplementary material for: Severity of Psychotic‐Like Experiences in Help‐Seeking German Adolescents: An Exploration of Psychosocial Predictors and Psychological Treatment Outcomes
Source: Early Interv Psychiatry. 2025 Aug 3;19(8):e70084. doi: 10.1111/eip.70084 (PMC12319175; doi:10.1111/eip.70084)
Supplement: Supplementary file 1 — Data S1: Diagnoses in the categories. [file EIP-19-0-s001.docx]

**Supplementary Material**

**Appendix 1.** Diagnoses in the Categories

| **Mood Disorders** | **Anxiety Disorders** | **Behavioural Disorders** |
| --- | --- | --- |
| F30 Manic episode | F40 Phobic disorders | F50 Eating disorders |
| F31 Bipolar affective disorder | F41 Other anxiety disorders | F51 Non-organic sleep disorders |
| F32 Depressive episode | F42 Obsessive-compulsive disorder | F52 Sexual dysfunctions not caused by an organic disorder or disease |
| F33 Recurrent depressive disorder | F43 Reactions to severe stress and adjustment disorders | F53 Postpartum psychological or behavioural disorders |
| F34 Persistent affective disorders | F44 Dissociative disorders | F54 Psychological or behavioural factors in diseases classified elsewhere |
| F38 Other affective disorders | F45 Somatoform disorders | F55 Harmful use of non-addictive substances |
| F39 Unspecified affective disorder | F48 Other neurotic disorders | F59 Unspecified behavioural abnormalities associated with physical disorders and factors |
|  |  | F90 Attention-deficit hyperactivity disorders |
|  |  | F91 Conduct disorders |
